# Differentiation of Cold Tolerance in an Artificial Population of a Mangrove Species, *Kandelia obovata*, Is Associated With Geographic Origins

**DOI:** 10.3389/fpls.2021.695746

**Published:** 2022-02-03

**Authors:** Wen-Xun Lu, Bing-Huang Zhang, Yuan-Ye Zhang, Sheng-Chang Yang

**Affiliations:** ^1^College of the Environment and Ecology, Xiamen University, Xiamen, China; ^2^School of Life Sciences, Peking University, Beijing, China

**Keywords:** adaptation, *Kandelia obovata*, low temperature, mangroves, genetic structure

## Abstract

Temperature is one of the climatic factors that shape the geographic distribution of plant populations. Mangroves are temperature-sensitive plants, and their distributions are severely limited by low temperatures. It is unknown, however, to what extent temperature contributes to their population differentiation and evolution. *Kandelia obovata* (Rhizophoraceae) is a mangrove species with high cold tolerance in the Northern Hemisphere. We investigated the phenotypic responses of an artificial population of *K. obovata*, with plants transplanted from different source populations, to extremely low temperatures during winter of 2015–2016 in Yueqing County (28°20′N), Zhejiang Province of China. Using two binary traits, “with/without leaves alive on the branches” and “with/without alive buds on the tips of branches,” we classified plants in this artificial population into strong, moderate and poor cold resistance groups. We further assessed the genetic diversity, structure and differentiation of these three groups, as well as five natural populations along a latitudinal gradient using ten nuclear and six plastid microsatellite markers. Microsatellite data revealed genetic differentiation among the natural populations along the latitudinal gradient. Molecular data indicated that the cold tolerance of three groups in the artificial population was associated with their geographic origins, and that the most cold-tolerant group came from the northernmost natural population. Our study thus indicates that natural populations of *K. obovata* may have evolved divergent capacity of cold tolerance.

## Introduction

Climate is one of the primary factors that shape the natural distribution, genetic and geographic structures of plant populations (MacArthur, 1984; Woodward, 1987; Etterson, 2004; Lomolino et al., 2010). Available evidence has shown that habitat preference, with a genetic basis, can be maintained by natural selection, which may eventually generate a series of genetic lineages corresponding to different environments (Lenormand, 2002; Kawecki and Ebert, 2004; Sork, 2016). Temperature, for example, can induce altitudinal and latitudinal differentiation of plant populations (Saxe et al., 2001; Jump et al., 2006; Halbritter et al., 2018). In addition, there are several case studies, such as on *Pseudotsuga menziesii* (Pinaceae) and *Cicer arietinum* (Fabaceae), that investigated the relationships of population genetic structure with phenotypic traits and with cold tolerance (Eckert et al., 2009; Sani et al., 2018).

Mangroves are temperature-sensitive plants. Both the annual coldest air temperature and the winter sea surface temperature can directly or indirectly affect mangroves (Chapman, 1977; Duke et al., 1998; Lovelock et al., 2016). Therefore, their distributions appear to be strongly limited by temperature in spite of some outliers, such as *Avicennia marina* var. *australasica* (Acanthaceae) in New Zealand (38°45′S), *Avicennia germinans* (Acanthaceae) in Bermuda (32°20′N), and *Kandelia obovata* in Japan (31°N), which may move from mangrove-rich regions to their latitudinal limits by the aids of warm ocean currents (Tomlinson, 2016). According to their adaptability to temperature, mangroves can be grouped into three thermal categories: thermophilic stenotopic species, thermophilic eurytopic species, and winter-resistant eurytopic species (Li and Lee, 1997; Hu et al., 2012). Since the 1980s, a series of studies have investigated the physiology, ecology, and genetics of cold resistance of mangroves (Lin et al., 1994; Yang and Lin, 1997, 1998; Yang et al., 1999, 2003; Wang et al., 2011; Chen et al., 2012, 2017; Zheng et al., 2012; Peng et al., 2015; Su et al., 2019). These studies revealed that morphological and physiological traits, such as tree defoliation, leaf photosynthesis rate, soluble sugar content, and leaf conductivity are indicators of the cold resistance of mangroves. Yang and Lin (1997) measured the cold-resistant ability of leaves of two mangrove species, *K. obovata* and *Aegiceras corniculatum* (Myrsinaceae), and found that their cold-resistant ability increased with decreasing air temperature. In a previous study, where the cold-hardiness of leaves of 16 mangrove species was measured by conductivity method, Yang and Lin (1998) found a latitudinal pattern of population differentiation, that is, a positive relationship between latitude and cold tolerance. Wu et al. (2018) examined the relationship between temperature and the distribution of mangrove species, and found that the species composition of a mangrove assemblage was significantly correlated with winter temperature. Yet, it is unknown to what extent temperature contributes to the population structure and evolution of mangrove species. We currently lack evidence that populations of a single mangrove species may differentiate under temperature control.

*Kandelia obovata* is a widely distributed mangrove species, and its distribution range contains the Ganges Delta, Burma, Southeast Asia, the Ryukyu Islands, and even southern Japan (Tomlinson, 2016). As a monoecious mangrove species, *K. obovata* is self-compatible, and depends on small insects for pollination (Tomlinson, 2016). It is regarded as one of the mangrove species with the highest cold tolerance in the Northern Hemisphere. Its natural northern range limit in China is Fuding City of Fujian Province (27°26′N) with an average winter temperature of 9.9°C (Lin, 1999; Wang et al., 2011). In 1957, *K. obovata* was successfully transplanted from Hainan Province to the Yueqing County (28°20′N) of Wenzhou City in Zhejiang Province for the first time (Mu et al., 2005). Subsequently, propagules of *K. obovata* from other areas, such as Fuding County in Fujian Province and Cangnan County in Zhejiang Province, were gradually introduced to Yueqing (Lin, 1999; Mu et al., 2005; Chen et al., 2019). Nowadays, *K. obovata* plants introduced from different areas and at different periods have survived in Yueqing, which forms a mixed artificial population (Zheng et al., 2016). In a study on the population genetics of *K. obovata* along the southeastern coast of China, Ruan et al. (2013) found significant differentiation between most population pairs using AFLP markers. Other than that, Yang and Lin (1998) demonstrated that the cold-hardiness of leaves in different *K. obovata* populations increased with increasing latitude. However, it is unknown whether the difference in cold resistance of *K. obovata* populations is associated with their genetic differentiation. From 2008 to 2016, several extreme cold climatic events occurred in South China, having a pervasive influence on mangroves (Chen et al., 2017; Su et al., 2019). Especially, a chilling event in the winter of 2015–2016 caused differing degrees of defoliation and bud withering in accessions of *K. obovata* that were introduced from different natural populations to Yueqing. This phenomenon suggests different adaptability of source populations of *K. obovata* to extremely low temperatures.

To understand whether the observed divergence of cold resistance has genetic underpinnings, we investigated the aforementioned artificially mixed population of *K. obovata* in a “common garden” condition in comparison with five natural populations along a latitudinal gradient. We analyzed phenotypic responses of *K. obovata* to coldness at individual levels in Yueqing. Besides, we analyzed the population genetic diversity, structure and differentiation of Yueqing as well as five natural populations using ten nuclear and six plastid microsatellite markers. Furthermore, we compared the cold tolerance with the genetic data in Yueqing, and traced the geographic origin of individuals in this artificial population. Finally, we test the hypothesis that cold tolerance of *K. obovata* may exhibit a latitudinal gradient.

## Materials and Methods

### Study Sites and Timing

**FIGURE 1:**
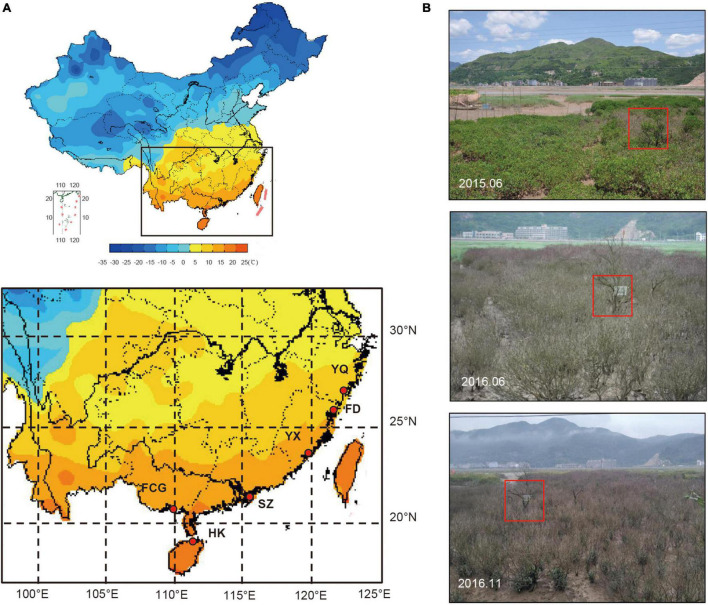
**(A)** Map of the study areas in China with details of the localities of six *K. obovata* populations and their average temperatures in January (from China Meteorological Data Sharing Service System). **(B)** *K. obovata* grown in YQ, from top to bottom, before the chilling event, 5 months after the chilling event, and 10 months after the event, respectively. The red boxes in the pictures indicate the same individual.

According to the available literature, Fuding (FD) was one of the known exact source populations for the Yueqing (YQ) artificial population (Chen et al., 2019). Populations from Hainan Island and the south of Fujian Province were also introduced to YQ (Li et al., 2001; Mu et al., 2005). Therefore, our experiments focused on the YQ artificially “mixed” population; in parallel, five populations along the southeast coast of China, including one exact source population (FD), two relatively accurate sources (Haikou, HK and Yunxiao, YX) and two possible provenances (Shenzhen, SZ and Fangchenggang, FCG) of YQ, were studied for comparison (Figure 1A and Table 1).

**TABLE 1:** Geographic information and meteorological characteristics of the areas where the studied populations are located.

Site	Abbr.	Number of samples	Latitude (N)	Longitude (E)	Area (hm^2^)	Mean annual temp. (°C)	Extreme low temp. (°C)	Tide type
Yueqing	YQ	160	28°20′	121°10′	0.24	18.7	−5.1	IST
Fuding	FD	23	27°16′	120°18′	100.1	18.8	−5.2	RST
Yunxiao	YX	24	23°95′	117°33′	133.5	21.7	0.0	RST
Shenzhen	SZ	21	22°32′	114°01′	304.0	23.0	1.9	IST
Fangchenggang	FCG	20	21°77′	108°35′	3057.3	22.7	2.8	RDT
Haikou	HK	26	19°57′	110°35′	3215.4	24.8	6.1	IDT

*Abbreviations of tide types: IST, irregular semi-diurnal tide; RST, regular semi-diurnal tide; RDT, regular diurnal tide; IDT, irregular diurnal tide.*

The chilling event that prompted this study occurred in YQ during the winter of 2015–2016 when the temperature fell to −5°C^1^. On June 17, 2016, we conducted the first survey and collected materials in YQ. Over a growing season approximately 10 months after the chilling event, our second “recovery” survey and materials sampling started on November 12, 2016 (Figure 1B). The investigation and sampling of five natural populations were conducted between August 20 and November 21, 2016.

### Sample Collection and Analysis of Morphological Traits

Almost all surviving adult individuals in YQ were sampled (126 samples for the first and 34 samples for the second investigation). From the five natural populations, 117 individuals were sampled with a distance of more than 10 m between each pair of individuals. In total, 277 individuals were sampled from six sites. Leaves were collected from each selected individual and then desiccated with silica gel, and then preserved at room temperature until DNA extraction. For the measurement of morphological traits, three mature leaves of each individual were used to measure leaf length (LL) and leaf width (LW), except for some individuals of the YQ population with a defoliation rate over 90%. In total, we obtained measurements of six morphological traits, e.g., plant height (PH), stem basal diameter (SBD), diameter at breast height (DBH), crown diameter (CD), leaf length (LL), and leaf width (LW; Supplementary Table 1; five traits for YQ and FD as their plant height was lower than 1.3 m rendering DBH missing data). Since the YQ population had experienced the chilling event, its individuals were recorded for several traits that may be directly related to cold stress, such as growth form (GF), defoliation percentage (DP), chilling damage to branches (CDB), and germination position (GP; Supplementary Table 1). All of the morphological traits measurement data used for subsequent analyses is listed in Supplementary Data Sheet 1. The average value, standard error (SE), and coefficient of variation (CV) of the above six traits and these cold-stress related traits were analyzed using Excel 2019 (Microsoft Inc., Redmond, WA, United States) and SPSS 22.0 software (SPSS Inc., Chicago, IL, United States). In addition, five quantitative variables (PH, SBD, CD, CDB, and DP) were standardized by subtracting the mean value, which was divided by the standard deviation to remove scale effects. A principal component analysis (PCA) was performed on those five quantitative traits using R version 4.0.0^2^ to depict the differences among different individuals in YQ.

### DNA Extraction and Screening of Microsatellite Fragments

Total genomic DNA was extracted from dried leaf samples using the Plant Genomic DNA Kit (Tiangen) following the standard protocol provided by the manufacturer. DNA extractions were checked for concentration and purity using NanoDrop 2000 spectrophotometer (Thermo Fisher Scientific), and subsequently diluted to a concentration of 5–20 ng/μL and stored at −20°C. For PCR amplification, 16 microsatellite markers (ten nSSR markers and six cpSSR markers) developed by previous studies were used (Supplementary Table 2; Sugaya et al., 2002; Islam et al., 2006, 2008), and PCR reactions were performed following the published protocol (Sugaya et al., 2002; Islam et al., 2006, 2008). Forward primers for PCR amplifications were fluorescence-labeled with HEX (green) or ROX (red). Amplification products were screened using capillary electrophoresis (CE) [Sangon Biotech (Shanghai) Co., Ltd.]. For samples that exhibited ambiguous bands, amplifications were repeated twice with sterilized water used as the negative control to exclude systemic error.

### Nuclear Simple Sequence Repeats Genetic Diversity and Differentiation

Although 160 samples were collected in YQ, there were 12 samples with missing data, and therefore a total of 148 individuals in YQ and 114 individuals in five natural populations (three samples with missing data were excluded) were included in the subsequent analysis. The simple sequence repeats (SSRs) data (both nSSR loci and plastid SSR loci) used for subsequent analyses is listed in Supplementary Data Sheet 2. For both YQ artificial population and five natural populations, GENEPOP 3.4 (Raymond and Rousset, 1995) was used to test for null alleles, the Hardy--Weinberg equilibrium (*HWE*) and for linkage disequilibrium (*LD*). The nSSR loci under selection were detected with BayeScan 2.0^3^. Both neutral loci and all loci were used to calculate genetic diversity, but only neutral loci were used to infer population structure. Genetic diversity parameters including the number of alleles (*Na*), number of effective alleles (*Ne*), Shannon diversity index (*I*), observed heterozygosity (*Ho*), expected heterozygosity (*He*), and unbiased expected heterozygosity (*uHe*) were calculated using GenAlEx 6.5 (Peakall and Smouse, 2012). Population pairwise *F*_*ST*_ was computed using Genetix 4.05 with 1,000 permutations to calculate the significance of *F*_*ST*_ (Belkhir et al., 1996-2004). The analysis of molecular variance (AMOVA) was performed to assess the distribution of genetic variance among different populations using Φ-statistics in GenAlEx 6.5 (Peakall and Smouse, 2012). Two AMOVA’s were conducted, one is to group individuals by sampling locality, and the other to group individuals by different cold resistance in YQ population. The potential structure of the populations (YQ and five natural populations) was inferred from a Bayesian approach using STRUCTURE 2.3.3 based on the admixture model with correlated allele frequencies between populations (Pritchard et al., 2000). For each simulated value of *K* (the number of clusters, varying from 1 to 10), 20 independent runs were performed with the Markov chain Monte Carlo (MCMC) run for 1,000,000 replications and a burn-in period of 100,000 interactions. The most likely number of *K* was determined following the method described by Evanno et al. (2005).

### Plastid Simple Sequence Repeats Genetic Diversity and Geographic Patterns

GenAlEx 6.5 was used to calculate genetic diversity parameters, the number of alleles (*Na*), number of effective alleles (*Ne*), Shannon diversity index (*I*), haplotype diversity (*h*), and unbiased diversity (*uh*) (Peakall and Smouse, 2012). AMOVA was performed to assess the distribution of genetic variance among different cold resistance groups in YQ using GenAlEx 6.5. Plastid haplotypes were analyzed using Arlequin 3.5 (Excoffier and Lischer, 2010), and the haplotype network was constructed with NETWORK 10.1^4^ using the Median Joining method. BAPS 6 was used with a spatial genetic mixture model to infer the geographic patterns of cpSSRs among different populations (Corander et al., 2008a,b). We ran the model for ten replicates with *K* ranging between 1 and 20 using the option of “clustering with linked loci” and chose the *K* with the highest log-likelihood. The presence of phylogeographic structure was determined using the ratio of *N*_*ST*_ (considering the mutational distances between haplotypes) and *G*_*ST*_ (depending on the frequencies of haplotypes) with 1,000 random permutations in Permut cpSSR 2.0 (Pons and Petit, 1996).

## Results

### Phenotypic Responses to Chilling Event

**FIGURE 2:**
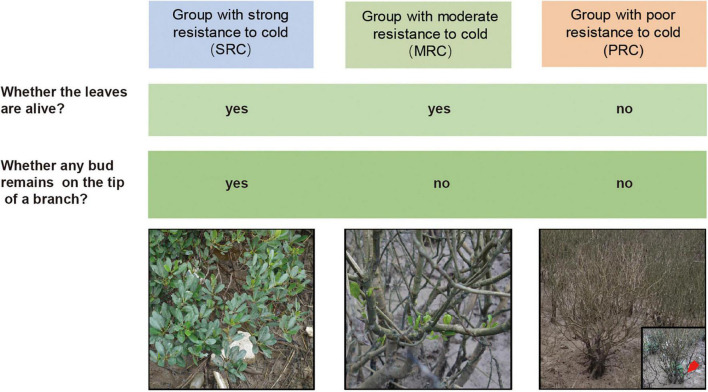
Criteria for classifying individuals into strong, moderate, and poor resistance to cold. Images of three groups of plants were taken after the chilling event in the winter of 2015–2016. The red arrow points to the newly grown branches of the “dead” individual during the second survey.

Based on the phenotypic response of individuals to chilling event in the YQ population during the winter of 2015–2016, we could classify them into three categories of cold resistance: the group with strong resistance to cold (SRC), the group with moderate resistance to cold (MRC) and the group with poor resistance to cold (PRC) (Figure 2). The results of PCA on five quantitative morphological traits also showed differentiation between the above three categories (Supplementary Figure 1). Further, we analyzed the traits of three categories in the YQ population in response to low temperature. The chilling damage to branches (estimated as the proportion of completely damaged branches) of the SRC group was 40%, which was much lower than the 89.7 and 100.0% estimated for MRC and PRC groups (Table 2). The defoliation percentage of SRC group was 57.1%, which was also much lower than the 96.1 and 100.0% estimated for the other two groups. However, certain “dead” individuals in the PRC group had new branches, and leaves grew out at the base of the trunk 10 months after the chilling event (Figure 2). Compared to the other two groups, the SRC group showed a relatively large variation of chilling damage to branches and defoliation percentage. Among all analyzed traits, the coefficient of variation was largest in plant height and stem basal diameter for all three cold-resistance groups (Table 2).

**TABLE 2:** Phenotypic traits of three groups with different cold resistance in the YQ population after the chilling event during the winter of 2015–2016.

	SRC			MRC			PRC			Total		
	Mean	SD	CV (%)	Mean	SD	CV (%)	Mean	SD	CV (%)	Mean	SD	CV (%)
PH (m)	1.25	0.41	33.65	1.80	0.52	28.8	1.23	0.51	41.21	1.59	0.56	35.64
SBD (cm)*^a^*	3.68	7.61	206.72	12.70	11.18	88.08	1.00	0.64	63.63	8.75	10.67	122.03
CD (m)	0.41	0.49	120.11	1.32	1.71	129.99	0.25	0.19	76.29	0.94	1.45	154.09
LL (cm)*^b^*	7.73	0.54	6.99	8.25	0.58	7.04	11.11	0.76	6.86	9.31	1.71	18.35
LW (cm)	3.78	0.29	7.63	3.90	0.33	8.40	5.15	0.34	6.65	4.40	0.73	16.61
CDB (%)	40.00	20.78	51.96	89.70	9.04	10.07	100.0	0.00	0.0	83.81	22.59	26.96
DP (%)	57.12	21.03	36.82	96.10	4.70	4.89	100.0	0.00	0.0	90.59	17.45	19.26

*SRC, group with strong resistance to cold; MRC, group with moderate resistance to cold; PRC, group with poor resistance to cold; PH, plant height; SBD, stem basal diameter; CD, crown diameter; LL, leaf length; LW, leaf width; CDB, chilling damage to branches; DP, defoliation percentage.*

*^a^SBD is the diameter of the thickest branch; individuals with poor resistance to cold (PRC) form more than one branch at the base of their stems.*

*^b^Because the response to the chilling event of each individual was extremely different, certain individuals did not have enough mature leaves for trait measurement, especially in MRC. Eventually, 238 leaves were available for analysis (77 leaves from SRC, 59 leaves from MRC, and 102 leaves from PRC).*

Recent studies have shown that leaf size was often correlated with mean annual temperature (Moles et al., 2014). To test this, we measured leaf sizes of individuals from mixed population YQ and from the five natural populations. Regarding the leaf trait analysis, certain individuals in YQ did not have enough leaves for measurement. Eventually, 238 leaves were available for analysis (77 leaves from SRC, 59 leaves from MRC, and 102 leaves from PRC). We found that SRC had the smallest leaves and PRC had the largest (Table 2). In parallel, we measured all six phenotypic traits in five natural populations and similarly found a gradual decrease in leaf size along latitude (Supplementary Table 3).

### Genetic Diversity, Variation and Structure Revealed by Nuclear Simple Sequence Repeats

Of the ten loci, the mean frequency of null alleles was 10%, ranging from 2.2 to 11.9%. Across the five natural populations, no signs of *LD* were found, while 10 out of 45 pairwise comparisons showed significant *LD* in YQ population (*p* < 0.01). Four nSSR loci (Kaca01, Kaca04, Kaca12, and Kcan004) deviated significantly from *HWE* (*p* < 0.01). One locus (Kcan011) was identified by BayeScan as an outlier and the remaining nine loci were considered neutral (Supplementary Table 4). In total, 176 alleles of 10 nuclear microsatellite loci were detected in six populations, and the number of alleles per locus (*Na*) ranged from 8 to 35, with a mean of 17.6 per locus. The genetic diversity parameters for each nSSR locus were shown in Supplementary Table 5.

Genetic diversity parameters of six populations are shown in Table 3. In general, genetic diversity of natural populations decreased with increasing latitude, except for population HK. As an artificial mixed population, various genetic diversity parameters of the artificial YQ population, including *Ne*, *Ho*, *He*, and *I*, were generally within the range of natural populations (Table 3). When Kcan011 was excluded, genetic diversity decreased slightly based on 9 neutral nSSR loci, but the pattern of genetic diversity among above populations did not change (Supplementary Table 6). In addition to the geographic grouping, three groups of *K. obovata* with different cold resistance in YQ population were analyzed based on 9 neutral nSSR loci and all nSSR loci separately. We found different levels of polymorphism at nuclear SSR loci: the MRC group had the highest genetic diversity, followed by the PRC group, and the SRC group displayed the lowest genetic diversity (Supplementary Tables 7, 8).

**TABLE 3:** Genetic diversities within each of the six populations of *K. obovata* based on ten nSSR loci and six cpSSR loci.

Pop.		*nSSR*						*cpSSR*				
	*N*	*Na*	*Ne*	*Ho*	*He*	*uHe*	*I*	*Na*	*Ne*	*I*	*h*	*uh*
YQ	148	10.700	4.890	0.586	0.686	0.689	1.620	1.667	1.264	0.201	0.116	0.119
FD	23	4.400	2.395	0.487	0.481	0.492	0.942	1.500	1.179	0.160	0.086	0.090
YX	24	8.500	4.744	0.625	0.725	0.740	1.625	1.667	1.287	0.289	0.181	0.189
SZ	21	9.000	5.385	0.643	0.734	0.752	1.714	1.667	1.481	0.358	0.231	0.243
FCG	20	9.400	5.607	0.660	0.755	0.775	1.776	1.833	1.353	0.356	0.224	0.236
HK	26	6.500	2.930	0.542	0.574	0.585	1.230	2.333	1.592	0.431	0.242	0.251

*N, number of individuals; Na, number of alleles; Ne, effective number of alleles; Ho, observed heterozygosity; He, expected heterozygosity; uHe, unbiased expected heterozygosity; I, Shannon’s index of diversity; h, diversity; uh, unbiased diversity. See Table 1 for population abbreviations.*

Genetic differentiation among six populations assessed by pairwise *F*_*ST*_ is listed in Table 4. The pairwise measure of differentiation in all populations was significant, but the degree of genetic differentiation among YX, SZ, and FCG populations was relatively low. AMOVA based on nuclear SSRs among five natural populations (Table 5) revealed that the genetic variation mainly existed within populations (72%), only 28% occurred among different populations (*p* < 0.001). Similarly, AMOVA based on nuclear microsatellites in YQ revealed that major genetic differences existed within different cold resistance groups (87%), and only 13% occurred among different groups (*p* < 0.001) (Supplementary Table 9).

**TABLE 4:** Population pairwise *F*_*ST*_ of the six populations of *K. obovata*.

	YQ	FD	YX	SZ	FCG	HK
YQ		+	+	+	+	+
FD	0.147		+	+	+	+
YX	0.077	0.208		+	+	+
SZ	0.099	0.189	0.075		+	+
FCG	0.132	0.241	0.096	0.051		+
HK	0.224	0.346	0.197	0.151	0.182	

*See Table 1 for population abbreviations. +, significant differences at p < 0.05.*

**TABLE 5:** Summary of the AMOVA results for five natural populations of *K. obovata*.

Source	d.f.	SS	MS	Estimated variance	Percentage %	Phi statistic	Value	*p*
Among populations	4	289.695	72.424	2.858	28%	PhiPR	0.278	<0.0010
Within populations	109	807.937	7.412	7.412	72%			
Total	113	1097.632		10.270	100%			

*d.f., degree of freedom; SS, sum of squared observations; MS, mean of squared observations; PhiPR, proportion of the total genetic variance that is due to the variance among populations within a region.*

**FIGURE 3:**
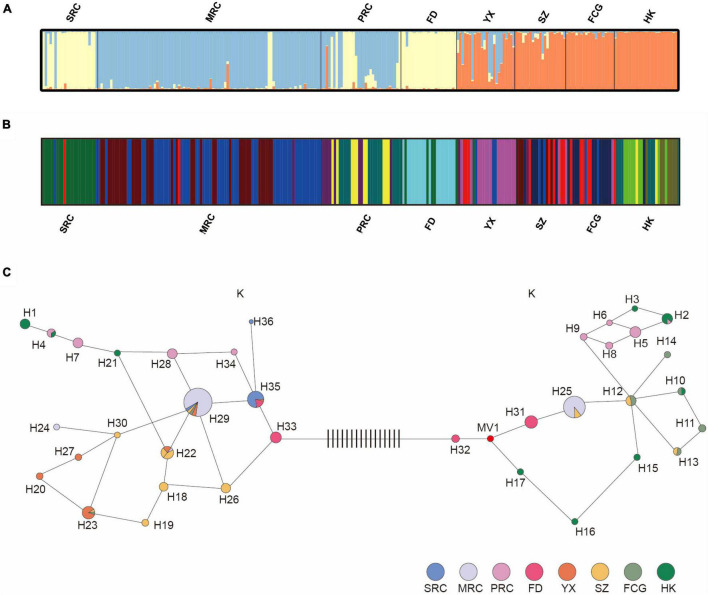
Estimated genetic structures of six populations based on nine nSSR loci **(A)** and on six cpSSR loci **(B)**. **(C)** Plastid haplotype network of YQ and five natural populations, each circle represents a haplotype, and the color within the circle represents the populations or groups that share that haplotype. Black vertical lines between haplotype 32 and 33 represent mutation steps. See Table 1 for population abbreviations.

The Bayesian clustering analysis for six populations using nine neutral loci indicated that *K* = 3 was the optimal parameter of their genetic structure (Figure 3A). These three groups were distributed among the six populations as follows: one group contained all individuals in FD and some individuals in YQ (there mainly, but not exclusively, in the SRC group); one group consisted of all individuals of populations HK and FCG, almost all individuals in SZ (with some admixture), and several admixed individuals YX; the last group was composed of few individuals in YX and most individuals in YQ (nearly all MRC and most PRC individuals). Consequently, there was no clear association between phenotypic and genetic variation, as phenotypic groups of different cold resistance in YQ did not correlate with genetic groups.

### Haplotype Diversity and Geographic Patterns Inferred From Plastid Simple Sequence Repeats

The genetic polymorphisms at the six plastid microsatellite loci were relatively low (Supplementary Table 10). Only 24 alleles were detected across six cpSSR loci in all populations with *Na* varying from 2 to 8, the genetic diversity *h* ranging from 0.008 to 0.719, and Shannon’s index *I* ranging from 0.025 to 1.516. Regarding plastid microsatellite polymorphisms based on collection sites, genetic diversity decreases with increasing latitude in natural populations, and these genetic diversity parameters of YQ population were also within the range of the natural populations (Table 3). Interestingly, genetic diversity similarly decreases with increasing cold resistance in YQ population. The highest plastid polymorphism was observed in PRC (*Na* = 2.000, *Ne* = 1.577, *h* = 0.219, *I* = 0.384) and the lowest was in SRC (*Na* = 1.500, *Ne* = 1.201, *h* = 0.122, *I* = 0.200) (Supplementary Table 7). AMOVA based on cpSSR in YQ revealed that the plastid microsatellites variation was 63% among different cold resistance groups and 37% within groups (*p* < 0.001), consistent with the haplotype sharing between different groups (Supplementary Table 11).

A total of 15 plastid microsatellite haplotypes were found across three phenotypic groups of the artificial population (Figure 3C and Supplementary Table 12). Only one haplotype (H29) was shared between SRC and MRC, and the most frequent haplotypes were different among the three groups, indicating different origins of these three groups. For example, the most frequent haplotype of SRC was H35 (87%), and that of MRC was H29 (59%). In total, 36 haplotypes were found in six populations, of which six were shared by the natural populations and the artificial population in YQ (Figure 3C and Supplementary Table 12). Haplotypes H2 and H4 found in the southernmost natural population HK were shared with PRC. Haplotypes H22, H25, and H29 found in the medium latitude populations, including YX, SZ, and FCG, were also found in MRC in YQ. The most frequent haplotype H35 in SRC was primarily observed in the northernmost natural population of FD.

The population structure analysis based on cpSSRs identified 12 different clusters through BAPS software (*K* = 12 was the optimal parameter, log-likelihood values = −352.618) (Figure 3B). In detail, in the YQ population, groups with different cold resistance each occupied different clusters, and there were almost no shared clusters between different groups. In natural populations, FD and HK occupied their own unique clusters, while there was cluster sharing among YX, SZ, and FCG. Interestingly, SRC in YQ population shared some clusters with population FD, MRC shared the same clusters with three populations (YX, SZ, and FCG), and PRC shared some clusters with population HK.

The plastid haplotype network using the Median Joining methods indicated that the 36 haplotypes were grouped into two major clusters (Figure 3C), separated by sixteen mutational steps. Cluster I consisted of 19 haplotypes, and cluster II consisted of 17 haplotypes. Interestingly, MRC and PRC appeared in both clusters, whereas SRC only contained haplotypes belonging to cluster I. In addition, FD and HK populations hardly shared haplotypes with other natural populations, indicating significant phylogeographic structure across the five populations of *K. obovata*. The presence of phylogeographic structure was also confirmed by *R*_*ST*_ (0.308) being significantly higher than *G*_*ST*_ (0.217) (*P* < 0.001).

## Discussion

Low temperature has been regarded as one of the primary factors affecting the latitudinal range limits of mangrove species (Lugo and Patterson-Zucca, 1977; Duke et al., 1998; Stuart et al., 2007; Ross et al., 2009; Tomlinson, 2016; Osland et al., 2017; Wu et al., 2018). Yang and Lin (1998) showed an increase of cold resistance within *K. obovata* along latitude. The chilling event that occurred during the winter of 2015–2016 in Yueqing provided us with an ideal opportunity to obtain data of phenotypic response of *K. obovata* to low temperature and to compare the cold-resistant capability between populations of different geographic origins.

Among several chilling injuries, defoliation is a typical phenotypic response found in mangroves (Ellis et al., 2006; Tomlinson, 2016). A previous study reported that plants of *K. obovata* in the Shacheng Bay (27°17′N, 120°18′E) displayed leaf scorch, defoliation, and bud withering after frost (Wang et al., 2011). However, another study showed no defoliation in *K. obovata* populations during the 2008 chilling event (Chen et al., 2017). In this study, we found that individuals of *K. obovata* in the artificially mixed population YQ could be divided into three different phenotypic groups according to their appearance of defoliation and bud withering (Figure 2). Furthermore, the reduced leaf length and width in the YQ population was associated with an increase of cold resistance, and the same trend was observed among natural populations along the latitudinal gradient, which may indicate that smaller leaf size was more adapted to low temperatures (Rosbakh et al., 2015). Nevertheless, the standard deviation and CV of phenotypic traits within each cold resistance type were relatively large, probably due to mixed individuals introduced from multiple natural populations since the 1950s.

Climate change can drive local adaptation of plants, especially trees (Petit and Hampe, 2006). The adaptive diversification of plant populations can be strongly shaped by historical and contemporary climatic factors (Turesson, 1925; Linhart and Grant, 1996; Etterson, 2004; Jordan et al., 2017; Cuervo-Alarcon et al., 2018). As an important selective agent, the temperature may affect the population adaptation of a species over altitude and latitude gradients (Saxe et al., 2001; Kitamura et al., 2020). Mangroves are temperature-sensitive plants, and thus low temperature may exert strong selective forces on natural populations. Temperature is likely a critical factor in determining mangrove community assemblage and species diversity (Yang and Lin, 1998; Wang et al., 2011; Ruan et al., 2013; Wu et al., 2018).

Despite that microsatellites are usually regarded as neutral genetic markers, they may also reflect population genetic composition shaped by natural selection (probably due to being linked to the selection-targeting loci), and thus, can be used for detecting ecological adaptation (Thibert-Plante and Hendry, 2010; Jay et al., 2012). Especially plastid genomes usually lack DNA recombination, so that neutral markers may be more likely linked to selection-targeting loci. Previous case studies, such as on oak, coast redwood and runner bean, demonstrated that plastid neutral makers clearly revealed genetic structure related to adaptation (Rodriguez et al., 2013; Yan et al., 2019; Breidenbach et al., 2020; Danusevičius et al., 2021). In the present study, the plastid microsatellites revealed genetic differentiation among three groups of different cold tolerance in YQ, the artificial population, and a significant geographical structure of haplotypes among natural populations along the latitudinal gradient. We found that the population at high latitude (FD) mainly shared haplotypes with the SRC group in YQ. Similarly, the population at low latitudes (HK) mainly shared haplotypes with the PRC phenotype group in YQ (Figure 3C and Supplementary Table 12). These results suggest that the cold resistance difference between SRC, MRC and PRC groups in the artificial populations may be explained by their origin from northern, mid-latitude and southern populations in nature. If plants in the artificial population represent an unbiased sample from the corresponding natural populations, our study indicates these natural populations are differentiated in cold resistance, which is likely the consequence of local adaption to different temperatures across latitudes.

Nevertheless, by using nuclear microsatellites we failed to detect a clear correlation between phenotypic and genetic patterns as revealed by the plastid markers. One reason could be that, concerning neutral genetic markers, plastid markers in this case may be more effective than nuclear markers in providing information of ecological adaptation due to the lack of DNA recombination in the plastome and thus stronger linkage to the selection-targeted loci. This assumes that at least some of the relevant loci are in the plastid genome, which requires further studies. A second reason may be the extent of gene flow. Previous studies reported that pollen dispersal distances are often long in tropical tree species, which depend on insects for pollination (Ward et al., 2005; Geng et al., 2008). In theory, natural selection is expected to decrease genetic diversity within a population while increasing the differentiations among populations. In contrast, strong gene flow may reduce divergence among populations while increasing variation within individual populations. Therefore, nuclear gene flow, including contributions from pollen flow over larger distances, may have blurred the imprint of natural selection on the population differentiation. Finally, another possible explanation is that we failed to collect the precise source populations of all individuals in the YQ population.

## Data Availability Statement

The original contributions presented in the study are included in the article/Supplementary Material, further inquiries can be directed to the corresponding author.

## Author Contributions

W-XL and S-CY conceived the idea, designed the experiments, and collected the plant materials. W-XL and B-HZ conducted the molecular work. W-XL performed the analyses and drafted the manuscript. W-XL, S-CY and Y-YZ revised the manuscript. All authors contributed to the study and approved the submitted version.

## Conflict of Interest

The authors declare that the research was conducted in the absence of any commercial or financial relationships that could be construed as a potential conflict of interest.

## Publisher’s Note

All claims expressed in this article are solely those of the authors and do not necessarily represent those of their affiliated organizations, or those of the publisher, the editors and the reviewers. Any product that may be evaluated in this article, or claim that may be made by its manufacturer, is not guaranteed or endorsed by the publisher.

## Footnotes

1
http://www.tianqihoubao.com/lishi/leqing.html

2
https://www.r-project.org/

3
http://cmpg.unibe.ch/software/BayeScan/download.html

4
https://www.fluxus-engineering.com
