# Supplementary material for: Differentiation of Cold Tolerance in an Artificial Population of a Mangrove Species, Kandelia obovata, Is Associated With Geographic Origins
Source: Front Plant Sci. 2022 Feb 3;12:695746. doi: 10.3389/fpls.2021.695746 (PMC8851163; doi:10.3389/fpls.2021.695746)
Supplement: Supplementary file 1 [file Presentation_1.pdf]

Supplementary Material

Supplementary Tables

Supplementary Table S1 | Ten morphological traits measured in this study.

Code	Trait[a]
PH	Plant height (m)
SBD	Stem basal diameter (cm)
DBH	Diameter at breast height (cm)
CD	Crown diameter (m)
LL[b]	Leaf length (cm)
LW	Leaf width (cm)
GF	Growth form: main stem branching or not
GP	Germination position: main stem base or middle part, or on the tip of a branch
CDB	Chilling damage to branches: proportion of completely damaged branches (no leaves)
DP	Defoliation percentage

aSix traits (PH, BD, DBH, CD, LL, and LW) were measured for individuals of all populations, and the other four traits were measured only for the samples of the YQ population.

bFor each of the samples, three mature leaves were selected to measure the leaf length and leaf width, except for YQ, where the defoliation of the selected tree was extremely severe.

Supplementary Table S2 | Information of ten nSSR and six cpSSR primer pairs.

Locus	repeat	Primer sequence (5'−3')	T_m(°C)
Kaca01	$(CT)_{14}(GT)_6$	F: ATTCACACTAGTCCACTTCTCCGC	60
(AB256021)		R: ACACACACACACAGAGAGAGAG	
Kaca04	$(GT)_{19}(CT)_6$	F: ATCGTAGCGCACGAATGCTTAATA	57
(AB256023)		R: AGAGAGAGAGAGACACACACAC	
Kaca05	$(AC)_6(TC)_5(AC)_{11}$	F: GTAGTGCGGTGAGGTTTATAGGAA	58
(AB256024)		R: ACACACACACACTCTCTCTCTC	
Kaca09	$(GA)_{14}(GT)_6$	F: TCAAAAGAGAAGGTATATCAGAGG	55
(AB256025)		R: ACACACACACACTCTCTCTCTC	
Kaca10	$(GT)_{14}(GA)_6$	F: TTTTTAGATCTGGCCGGCACGTGC	62
(AB256026)		R: TCTCTCTCTCTCACACACACAC	
Kaca12	$(TC)_6(AC)_7$	F: GGTTCATGCTGGCCAATTTGGCAA	57
(AB256027)		R: TCTCTCTCTCTCACACACACAC	
Kcan004	$(CT)_{35}$	F: TGAACTGAAAAACACTCGAAAAT	50
(AB063360)		R: GCGCCCCTAATTAATGCTT	
Kcan009	$(GA)_{20}$	F: CCCGAACTGATGCAGATTCA	50
(AB063361)		R: AGGATGGGTCTTTACAGGTTATTT	
Kcan011	$(CT)_{34}$	F: AGCCACTCAGGTGTTCTATG	50
(AB063362)		R: CAGGTCTCATGGCTGTGTCC	
Kcan034	$(AG)_{26}$	F: CAGAAGCAGCAAGTAAGGAA	50
(AB063363)		R: GAAGAACGTGAAGACAGTGA	
Kacacp07	$A_8(GA)_9$	F: U19-TTAACTATTAACGTGTCATTC	54
(AB305115)		R: CCTCTCATGGTTCTCGGACAA	
Kacacp11	A_9	F: U19-TGCGACTATCTCCACTATA	53
(AB305117)		R: CCATAGAAGAAAGTATCTAGG	
Kacacp12	A_{10}	F: U19-AGATAGTTACCTTTCAGAA	53
(AB305118)		R: CGGCTCCGCAGTTAATTCTTT	
Kacacp15	$A_8(GA)_9$	F: U19-AACGTGTCAGCTCCTTTAT	53
(AB305120)		R: TAGAAAACATTCCAAGTCCTG	
Kacacp16	A_{12}	F: U19-GCTCAAATGTTTTCTATCTTT	52
(AB305121)		R: CCATTCGATTCTTTCACAAGC	
Kacacp17	$T_9(GT)_{10}$	F: U19-GTGAATCGTTAACGAAAAGAG	52
(AB305122)		R: CGACAACAAGTAAATTGATAG	

Supplementary Table S3 | Phenotypic traits of individuals in five natural populations. Phenotypic traits are abbreviated as follows: PH, plant height; SBD, stem basal diameter; DBH, diameter at breast height; CD, crown diameter; LL, leaf length; LW, leaf width.

Pop.	PH/m	SBD/cm	DBH/cm	CD/m	LL/cm	LW/cm
FD	1.82±0.51	4.36±1.29	3.48±0.38	0.99±0.37	8.05±0.91	3.97±0.40
YX	4.05±1.02	13.37±4.22	6.30±2.33	1.35±0.33	9.22±1.18	4.26±0.54
SZ	6.10±0.22	18.50±2.76	8.97±0.46	3.15±0.57	8.97±1.18	3.50±0.52
FCG	2.49±0.25	6.24±0.82	3.93±0.55	3.12±0.63	9.34±1.43	4.58±0.75
HK	5.53±0.99	10.60±3.23	6.26±1.81	2.77±0.54	9.97±1.54	4.44±0.81

Supplementary Table S4 | Results of tests for neutral evolution done with Bayescan 2.0. Prob, the posterior probability for the model including selection; Log10(PO), the logarithm of Posterior Odds to base 10 for the model including selection; qval, the q-value for the model including selection; alpha, the estimated alpha coefficient indicating the strength and direction of selection; Fst, the Fst coefficient averaged over populations.

Locus	Prob	Log10(PO)	qval	alpha	Fst
Kaca01	0.571114	0.124381	0.259585	-0.36006	0.12569
Kaca04	0.029006	-1.5247	0.73313	0.002374	0.16338
kaca05	0.030006	-1.5096	0.7067	-0.00508	0.16245
Kaca09	0.071214	-1.1153	0.57788	0.031002	0.16742
Kaca10	0.030006	-1.5096	0.7067	0.005592	0.16373
Kaca12	0.65013	0.26909	0.17493	0.51319	0.23804
Kcan004	0.14923	-0.75596	0.40738	-0.07286	0.1548
Kcan009	0.047009	-1.3069	0.63147	0.003965	0.16368
Kcan011	1	**1000**	**0**	-1.1455	0.064067
Kcan034	0.091018	-0.99943	0.5077	-0.04243	0.15835

Supplementary Table S5 | Genetic diversity of ten nSSRs in all populations. Abbreviations: *Na*, number of alleles; *Ne*, effective number of alleles; *Ho*, observed heterozygosity; *He*, expected heterozygosity; *uHe*, unbiased expected heterozygosity; *I*, Shannon's index of diversity.

Locus	*Na*	*Ne*	*Ho*	*He*	*uHe*	*I*
Kaca01	24	6.527	0.630	0.847	0.848	2.241
Kaca04	12	4.213	0.588	0.763	0.764	1.671
kaca05	17	5.315	0.664	0.812	0.813	1.989
Kaca09	8	1.814	0.328	0.449	0.450	0.934
Kaca10	15	8.779	0.882	0.886	0.888	2.347
Kaca12	8	2.891	0.099	0.654	0.655	1.361
Kcan004	31	8.118	0.702	0.877	0.878	2.541
Kcan009	10	2.070	0.412	0.517	0.518	0.998
Kcan011	35	14.683	0.901	0.932	0.934	2.992
Kcan034	16	5.106	0.664	0.804	0.806	1.867

Supplementary Table S6 | The genetic diversity parameters of nine neutral nSSR loci in all populations. Abbreviations: *N*, number of individuals; *Na*, number of alleles; *Ne*, effective number of alleles; *Ho*, observed heterozygosity; *He*, expected heterozygosity; *uHe*, unbiased expected heterozygosity; *I*, Shannon's index of diversity;

Pop	N	*Na*	*Ne*	*Ho*	*He*	*uHe*	*I*
YQ	148	9.333	3.929	0.549	0.660	0.662	1.488
FD	23	4.333	2.270	0.444	0.455	0.465	0.895
YX	24	7.222	3.952	0.593	0.704	0.718	1.503
SZ	21	7.556	4.453	0.603	0.713	0.730	1.588
FCG	20	8.556	4.698	0.628	0.736	0.755	1.672
HK	26	6.111	2.915	0.530	0.562	0.573	1.190

Supplementary Table S7 | The genetic diversity parameters for the three groups of *K. obovata* with different cold resistance in YQ based on ten nSSR loci. Abbreviations: *N*, number of individuals; *Na*, number of alleles; *Ne*, effective number of alleles; *Ho*, observed heterozygosity; *He*, expected heterozygosity; *uHe*, unbiased expected heterozygosity; *I*, Shannon's index of diversity; *h*, diversity; *uh*, unbiased diversity.

Group		*nSSR*						*cpSSR*				
	N	*Na*	*Ne*	*Ho*	*He*	*uHe*	*I*	*Na*	*Ne*	*I*	*h*	*uh*
SRC	23	5.100	2.800	0.457	0.556	0.569	1.113	1.333	1.051	0.078	0.039	0.041
MRC	92	9.100	4.712	0.624	0.675	0.679	1.541	1.667	1.163	0.142	0.090	0.091
PRC	33	8.100	3.807	0.573	0.651	0.661	1.459	2.000	1.557	0.384	0.219	0.225

Supplementary Table S8 | The genetic diversity parameters for the three groups of *K. obovata* with different cold resistance in YQ based on nine neutral nSSR loci. Abbreviations: *N*, number of individuals; *Na*, number of alleles; *Ne*, effective number of alleles; *Ho*, observed heterozygosity; *He*, expected heterozygosity; *uHe*, unbiased expected heterozygosity; *I*, Shannon's index of diversity;

Group	*N*	*Na*	*Ne*	*Ho*	*He*	*uHe*	*I*
SRC	23	4.556	2.496	0.396	0.527	0.539	1.024
MRC	92	7.778	3.559	0.586	0.646	0.650	1.396
PRC	33	7.222	3.428	0.552	0.628	0.637	1.363

Supplementary Table S9 | Summary of the AMOVA results for three groups of *K. obovata* with different cold resistance in YQ population base on nSSRs. Abbreviations: d.f., degree of freedom; SS, sum of squared observations; MS, mean of squared observations; PhiPT, proportion of the total genetic variance that is due to the variance among individuals within a variant.

Source	d.f.	SS	MS	Estimated Variance	Percentage %	Phi Statistic	Value	*p*
Among different groups with different cold resistance	2	103.917	51.959	1.118	13	PhiPT	0.133	0.00
Within groups	145	1059.873	7.309	7.309	87			
Total	147	1163.791		8.427	100			

Supplementary Table S10 | Genetic diversity of the selected six cpSSRs in all populations. *Na*, number of alleles; *Ne*, effective number of alleles; *I*, Shannon's index of diversity; *h*, diversity; *uh*, unbiased diversity.

Primer pairs	*Na*	*Ne*	*I*	*h*	*uh*
CP07	8	3.554	1.516	0.719	0.721
CP11	2	1.008	0.025	0.008	0.008
CP12	7	2.018	0.833	0.504	0.506
CP15	2	1.008	0.025	0.008	0.008
CP16	2	1.071	0.150	0.066	0.067
CP17	3	1.883	0.812	0.469	0.471

Supplementary Table S11 | Summary of the AMOVA results for three groups of *K. obovata* with different cold resistance in YQ population base on cpSSRs. Abbreviations: d.f., degree of freedom; SS, sum of squared observations; MS, mean of squared observations; PhiRT, proportion of the total genetic variance that is due to the variance between regions; PhiPR, proportion of the total genetic variance that is due to the variance among populations within a region.

Source	d.f.	SS	MS	Estimated Variance	Percentage %	Phi Statistic	Value	*p*
Among different groups with different cold resistance	2	47.524	23.762	0.586	63	PhiPT	0.633	0.000
Within groups	145	49.212	0.339	0.339	37			
Total	147	96.736		0.926	100			

Supplementary Table S12 | Details of haplotypes detected in YQ and five natural populations based on cpSSRs.

Haplotype	Combination						Distribution								Frequency (%)
	CP07	CP11	CP12	CP15	CP16	CP17	SRC	MRC	PRC	FD	YX	SZ	FCG	HK	
H1	127	179	195	112	130	161	0	0	0	0	0	0	0	9	3.435
H2	129	179	167	112	130	161	0	0	1	0	0	0	0	6	2.672
H3	129	179	167	112	130	163	0	0	0	0	0	0	0	1	0.382
H4	129	179	195	112	130	161	0	0	3	0	0	0	0	2	1.908
H5	131	179	167	112	130	161	0	0	9	0	0	0	0	0	3.435
H6	131	179	167	112	130	163	0	0	1	0	0	0	0	0	0.382
H7	131	179	195	112	130	161	0	0	5	0	0	0	0	0	1.908
H8	133	179	167	112	130	161	0	0	3	0	0	0	0	0	1.145
H9	133	179	167	112	130	163	0	0	1	0	0	0	0	0	0.382
H10	141	179	167	112	128	163	0	0	0	0	0	0	1	1	0.763
H11	141	179	167	112	128	165	0	0	0	0	0	0	2	0	0.763
H12	141	179	167	112	130	163	0	0	0	0	0	5	6	0	4.199
H13	141	179	167	112	130	165	0	0	0	0	0	1	1	0	0.763
H14	141	179	167	114	130	163	0	0	0	0	0	0	1	0	0.382
H15	141	179	169	112	130	161	0	0	0	0	0	0	0	1	0.382
H16	141	179	171	112	130	161	0	0	0	0	0	0	0	1	0.382
H17	141	179	173	112	130	161	0	0	0	0	0	0	0	1	0.382
H18	141	179	193	112	130	163	0	0	0	0	0	1	0	0	0.382
H19	141	179	193	112	130	165	0	0	0	0	0	1	0	0	0.382
H20	141	179	195	112	128	165	0	0	0	0	4	0	0	0	1.527
H21	141	179	195	112	130	161	0	0	0	0	0	0	0	4	1.527

H22	141	179	195	112	130	163	0	1	0	0	3	4	6	0	5.344
H23	141	179	195	112	130	165	0	0	0	0	12	1	1	0	5.344
H24	143	173	195	112	130	165	0	1	0	0	0	0	0	0	0.382
H25	143	179	167	112	130	163	0	36	0	0	0	4	0	0	16.794
H26	143	179	193	112	130	163	0	0	0	0	0	1	0	0	0.382
H27	143	179	195	112	128	165	0	0	0	0	1	0	0	0	0.382
H28	143	179	195	112	130	161	0	0	8	0	0	0	0	0	3.053
H29	143	179	195	112	130	163	2	54	0	0	3	2	2	0	24.046
H30	143	179	195	112	130	165	0	0	0	0	0	1	0	0	0.382
H31	145	179	167	112	130	163	0	0	0	15	0	0	0	0	5.725
H32	145	179	177	112	130	163	0	0	0	1	0	0	0	0	0.382
H33	145	179	193	112	130	163	0	0	0	2	0	0	0	0	0.763
H34	145	179	195	112	130	161	0	0	2	0	0	0	0	0	0.763
H35	145	179	195	112	130	163	20	0	0	5	1	0	0	0	8.397
H36	147	179	195	112	130	163	1	0	0	0	0	0	0	0	0.382

See Table 1 for populations abbreviations

Supplementary Figures

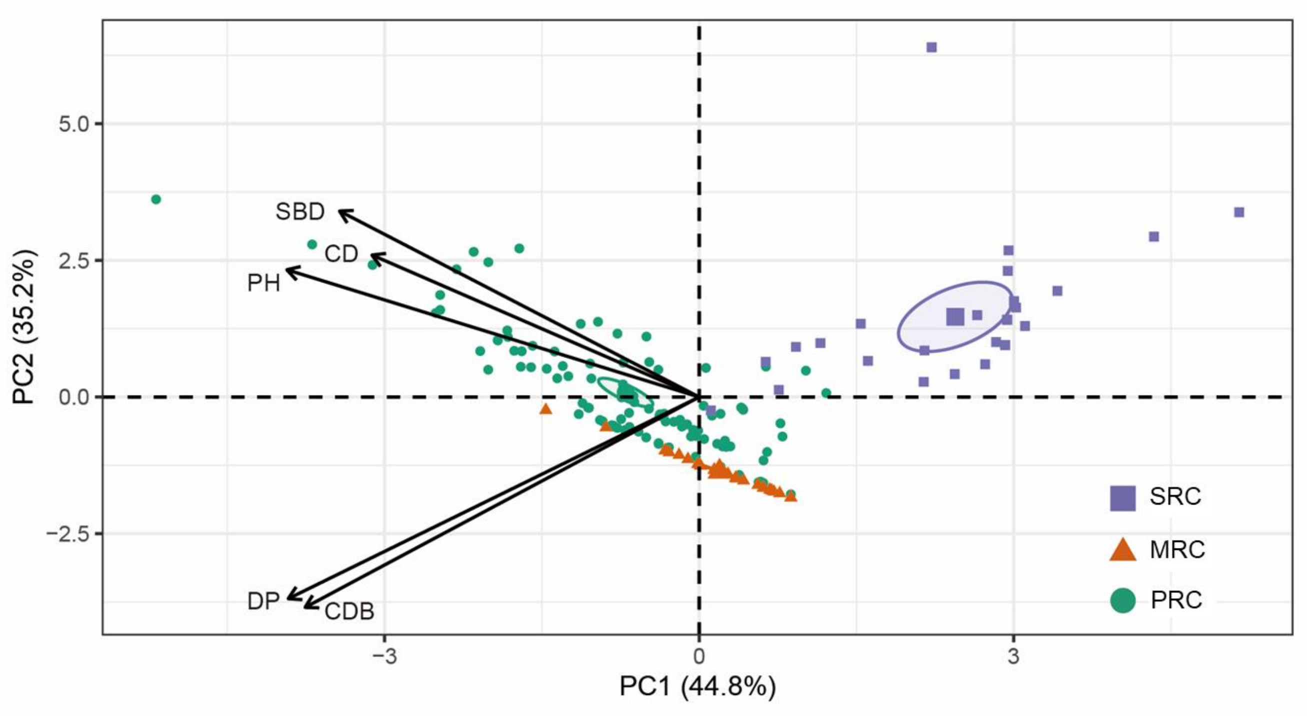

Supplementary Figure S1. Principal component analyses for the YQ population based on five morphological traits. Abbreviations: SRC, individuals with strong resistance to cold; MRC, individuals with moderate resistance to cold; PRC, individuals with poor resistance to cold; PH, plant height; SBD, stem basal diameter; CD, crown diameter; CDB, chilling damage to branches; DP, defoliation percentage.
